# Coping Strategies and Inflammatory Bowel Disease: A Narrative Review

**DOI:** 10.3390/jcm13061630

**Published:** 2024-03-12

**Authors:** Stefan L. Popa, Mihaela Fadgyas Stanculete, Simona Grad, Vlad Dumitru Brata, Traian-Adrian Duse, Andrei-Vlad Badulescu, Raquel-Vanessa Dragan, Paolo Bottalico, Cristina Pop, Abdulrahman Ismaiel, Daria-Claudia Turtoi, Dinu Iuliu Dumitrascu, Cristina Pojoga, Claudia Gherman, Liliana David

**Affiliations:** 1 2nd Department of Internal Medicine, “Iuliu Hatieganu” University of Medicine and Pharmacy, 400012 Cluj-Napoca, Romania; popa.stefan@umfcluj.ro (S.L.P.); costinsimona_m@yahoo.com (S.G.); abdulrahman.ismaiel@yahoo.com (A.I.); lilidavid2007@yahoo.com (L.D.); 2 Department of Neurosciences, Discipline of Psychiatry and Pediatric Psychiatry, “Iuliu Hatieganu” University of Medicine and Pharmacy, 400012 Cluj-Napoca, Romania; 3 Faculty of Medicine, “Iuliu Hatieganu” University of Medicine and Pharmacy, 400012 Cluj-Napoca, Romania; brata_vlad@yahoo.com (V.D.B.); adrianduse@yahoo.com (T.-A.D.); badulescu.andreivlad@elearn.umfcluj.ro (A.-V.B.); raqueldragan@yahoo.com (R.-V.D.); turtoidariaclaudia@gmail.com (D.-C.T.); 4 Department of Medical Sciences, University of Turin, 10126 Turin, Italy; paolo.bottalico@edu.unito.it; 5 Department of Pharmacology, Physiology, and Pathophysiology, Faculty of Pharmacy, “Iuliu Hatieganu” University of Medicine and Pharmacy, 400012 Cluj-Napoca, Romania; cristina.pop.farmacologie@gmail.com; 6 Department of Anatomy, “Iuliu Hatieganu” University of Medicine and Pharmacy, 400012 Cluj-Napoca, Romania; d.dumitrascu@yahoo.com; 7 Regional Institute of Gastroenterology and Hepatology, UBB Med., Babes Bolyai University, 400015 Cluj-Napoca, Romania; cristinapojoga@yahoo.com; 8 Department of Surgery, County Hospital, “Iuliu Hatieganu” University of Medicine and Pharmacy, 400012 Cluj-Napoca, Romania; gherman.claudia@umfcluj.ro

**Keywords:** inflammatory bowel disease, coping strategies, health related quality of life, psychosocial factors

## Abstract

**Background**: Coping strategies play a crucial role in managing inflammatory bowel disease (IBD), influencing both health-related quality of life (HRQoL) and psychological well-being. This study systematically reviews the available literature to analyze coping mechanisms in IBD populations and their impact. **Methods**: Relevant English-language studies published until 2023 were identified through a comprehensive search of PubMed, EMBASE, EBSCOhost, and Cochrane Library. After applying inclusion and exclusion criteria, 57 articles underwent full analysis. **Results**: The findings highlight the diversity of coping strategies used by individuals with IBD and emphasize the need for a nuanced approach considering factors like disease severity, duration, and individual characteristics. This review underlines the influence of coping mechanisms on QoL and indicates their potential to aid IBD management and rehabilitation. **Conclusions**: This study underscores the value of investigating coping strategies to promote better outcomes for individuals with IBD. Future research should explore personalized interventions that address the heterogeneity of the IBD population.

## 1. Introduction

Inflammatory bowel disease (IBD) is a multifactorial condition with an annual incidence of 396 cases per 100,000 individuals, resulting in over 700,000 physician visits and 100,000 hospitalizations [1,2,3]. It is classified as either ulcerative colitis (UC), which affects the colonic mucosa, or Crohn’s disease (CD), which causes transmural lesions in all segments of the gastrointestinal tract [4]. The etiology of IBD is believed to result from a complex interplay between genetic background, mucosal barrier dysfunction, disturbances in the gastrointestinal microbiota, dysregulated immune responses, and environmental factors such as diet, air pollution, smoking, drugs, geography, and psychosocial stress [4,5,6,7].

Despite significant advances in therapeutic approaches, up to 40% of patients with IBD in clinical trials are unresponsive to biological therapy, and up to 10–20% of patients in clinical series remain refractory to treatment [8]. Patient education regarding diet, exercise, and psychosocial factors has been shown to have a positive impact on IBD management [9,10].

The impact of IBD extends beyond its physiological manifestations and encompasses significant psychosocial dimensions that warrant systematic exploration. The psychosocial consequences of chronic illnesses have gained increasing attention in recent years. Recognizing the intricate interplay between psychological factors and disease progression is crucial for a comprehensive understanding of patient experiences and outcomes. Psychological stress, often implicated as a precipitating factor in IBD exacerbations, highlights the importance of investigating coping strategies as a critical aspect of disease management [10].

The unpredictable nature of IBD can drastically impact an individual’s productivity both at work and in their personal lives. Flare-ups, frequent restroom visits, fatigue, and pain can lead to missed workdays, reduced capacity, and difficulty meeting deadlines.

The integration of psychosocial assessments into routine clinical practice is a critical component of patient-centered care for individuals with chronic illnesses. Recognizing the diverse coping patterns exhibited by IBD patients is essential for developing tailored interventions that address individual needs. Adaptive coping mechanisms, including problem-solving strategies, seeking social support, and cultivating a positive cognitive outlook, have been proposed to mitigate the psychological burden of IBD [10]. Conversely, maladaptive coping strategies, such as avoidance and denial, may contribute to increased stress levels and the exacerbation of symptomatology [10].

Methodological rigor in examining coping strategies is crucial for establishing evidence-based relationships between psychological stress, coping strategies, and treatment efficacy. Therefore, this review aims to investigate the relationship between coping strategies and IBD. By synthesizing the existing evidence, we aimed to provide a foundation for future research and inform the development of tailored interventions that enhance the overall well-being of patients with IBDs.

## 2. Materials and Methods

### 2.1. Study Design

This study employs a narrative review design to synthesize the existing literature on the relationship between coping mechanisms and inflammatory bowel disease (IBD). Narrative reviews offer a broader and more interpretive approach compared to systematic reviews, aiming to identify patterns, themes, and potential contradictions within a body of research.

### 2.2. Search Strategy

Databases: A comprehensive search of the literature was conducted in the following electronic databases: PubMed, EMBASE, EBSCOhost, and Cochrane Library.

Search Terms: The search string combined terms related to IBD (“inflammatory bowel disease”, “Crohn’s disease”, “ulcerative colitis”) with concepts of coping (“coping mechanisms”, “coping strategies”, “adaptation”, “resilience”). Additional keywords may have included quality of life.

Date Range: The search was limited to studies published from their commencement until December 2023 to capture the most recent research advancements.

Language: Searches were restricted to English language publications.

### 2.3. Study Selection:

Inclusion Criteria: Studies were included if they met the following criteria: they examined the relationship between coping mechanisms and IBD in human populations, utilized qualitative, quantitative, or mixed-method designs, and were published in peer-reviewed journals.

Exclusion Criteria: Studies were excluded if they focused solely on pharmacological or surgical interventions for IBD, primarily discussed coping in general without a specific focus on IBD, and were review articles, case reports, or commentaries without original data.

### 2.4. Data Extraction and Analysis

Two reviewers independently (L.D. and S.L.P.) screened the titles and abstracts of the identified studies. The full-text articles of potentially relevant studies were assessed for their eligibility against the inclusion/exclusion criteria. In addition, a manual search of the reference lists of relevant articles was conducted to reduce the risk of bias and to identify any overlooked publications related to the review topic. If an article was determined to be eligible for inclusion in the evaluation but the full text could not be located, an email was sent to the authors requesting the full text. Eligible papers were evaluated, and data extraction was completed by the same reviewers.

A standardized data extraction form was used to collect the following information from each included study: the author(s), year of publication, study design, sample characteristics, measures of coping, IBD-related outcomes, and key findings.

Due to the anticipated heterogeneity among studies, a thematic synthesis approach was used to analyze the extracted data. Recurring themes and patterns related to coping mechanisms and IBD outcomes were identified and discussed.

### 2.5. Limitations

The potential limitations of this narrative review include the lack of formal quality assessment tools and meta-analysis, which are common in systematic reviews. It is important to address how these might impact the interpretation of our findings.

## 3. Results

The initial search yielded 798 articles in PubMed, 480 in EMBASE, 234 in EBSCOhost, and 72 in Cochrane Library. After applying human filters to the electronic databases, 157 publications were obtained. Subsequently, all filters were applied, and 97 studies were retained. Ultimately, 57 papers were included in this review (Table 1).

### 3.1. Coping Strategies and Quality of Life

The health-related quality of life (HRQoL) of individuals with CD and UC is influenced by various factors. These include disease activity, severity, and complications, such as strictures, fistulas, and abscesses, all of which can negatively impact QoL [17]. Effective coping strategies, such as medication adherence, stress management, and seeking support, can help individuals with IBD manage their symptom burden and enhance their psychological well-being, thereby positively impacting their HRQoL. However, the unpredictable nature of IBD and its potential for complications pose challenges to HRQoL.

Many studies examine the relationship between health-related quality of life (HRQoL) and coping strategies, demonstrating a statistically significant correlation between the two variables [11,12,13,14,15,17,19,20,22,23,24,26,29,30,32,36,37,38,40,41,42,44,45,48,49,55,56,58,61,62,66]. Specifically, maladaptive coping strategies, such as acceptance–resignation, negative religious coping, catastrophizing, passive coping, low acceptance, perceived control, decreased cognitive flexibility, and emotion-focused coping, were all associated with lower HRQoL.

Chao et al. found that both disease activity and maladaptive coping negatively affected HRQoL [13]. Patients with higher self-efficacy were more likely to report the use of adaptive coping strategies. In multivariate analysis, female patients, those with clinically active disease, and those with the structuring CD phenotype were at an increased risk of experiencing reduced productivity. Conversely, individuals with higher self-efficacy have a reduced risk of productivity loss. Clinical disease activity and maladaptive coping have been linked to higher levels of anxiety.

Kantidakis et al. found that illness perception served as a mediator in the relationship between IBD symptoms, psychological distress, and HRQoL. Psychological variables, including maladaptive and adaptive coping, mindfulness, and self-efficacy, were identified as partial mediators between illness perceptions and distress, with maladaptive coping being the most significant contributor to this variability [20].

A case–control study involving 70 Romanian patients with inflammatory bowel disease (IBD) and 70 healthy controls revealed a significant difference in stress levels, as measured by the Perceived Stress Scale, between the two groups with IBD patients exhibiting higher levels of stress. Furthermore, the IBD group displayed lower QoL scores in all domains of the SF-36 assessment except for emotional role functioning. However, the analysis of covariance for SF-36 domain scores and various clinical characteristics did not yield any significant results. The COPE instrument was used to analyze coping strategies in the IBD group, which revealed the highest scores for problem-focused and emotion-focused coping strategies, while lower scores were observed for maladaptive coping and alcohol/substance use [66].

Several studies included in this review explore the psychological factors that impact how patients with inflammatory bowel disease cope with their condition. A study by Iglesias-Rey et al. found that higher levels of anxiety were associated with a greater frequency of emotion-focused coping, while higher levels of depression were linked to a greater frequency of escape–avoidance strategies [18].

Resilience in individuals with IBD involves the ability to adapt to the challenges posed by the disease, including symptom flares, treatment side effects, and psychosocial stressors. It encompasses psychological flexibility, problem-solving skills, and a capacity to maintain a sense of well-being despite adversity [61].

In a preliminary investigation focusing on IBD stoma patients, Knowles et al. (2021) [20] found a significant relationship between maladaptive coping strategies and increased levels of anxiety and depressive symptoms. Subsequently, Sirois et al. conducted a longitudinal study to analyze the psychological profiles of individuals with inflammatory bowel disease, particularly those exhibiting thriving, resilience, and loss. The results indicated that individuals who experienced a decline in life satisfaction reported higher levels of depressive symptoms than those in the resilience and thriving groups. Furthermore, individuals in the loss category showed lower coping efficacy, lower illness acceptance, reduced perceived social support, and more depressive symptoms than those in the resilience or thriving groups. Sheehan et al. also found that a higher IBD self-efficacy score was associated with fewer systemic symptoms, a reduced need for daily coping strategies, and diminished emotional impact.

Wang et al. revealed a decrease in psychological resilience among individuals diagnosed with inflammatory bowel disease (IBD). Furthermore, their findings demonstrated a direct correlation between their mindfulness level, psychological resilience, and overall well-being, suggesting that increased mindfulness and stronger psychological resilience contribute to a greater sense of happiness. The results of this study imply that mindfulness levels not only exert a direct impact on overall well-being but also regulate it indirectly through the mediating variable of psychological resilience [42].

The interplay between coping mechanisms and HRQoL in individuals with IBD underscores the significance of adaptive coping strategies in promoting positive QoL outcomes. Effective coping strategies, such as problem solving, seeking social support, and fostering resilience, are instrumental in mitigating the impact of IBD symptoms and psychosocial stressors, ultimately contributing to enhanced well-being and satisfaction with life despite the challenges posed by the disease.

### 3.2. Problem-Focused Coping versus Emotion-Focused Coping

The use of active coping strategies, which involve taking direct action to address stressors and solve problems, has been linked to better disease management and quality of life in individuals with IBD. These strategies can enhance adherence to treatment regimens and self-care practices, leading to reduced disease activity and fewer complications.

Problem-focused coping involves the active monitoring of symptoms, disease activity, and treatment responses to identify patterns, triggers, and changes in health status. Keeping symptom diaries, tracking bowel movements, and using disease activity indices (e.g., Crohn’s Disease Activity Index and Ulcerative Colitis Disease Activity Index) can facilitate communication with healthcare providers, inform treatment decisions, and empower individuals to take an active role in managing their health.

While problem-focused coping strategies, such as seeking information and adhering to treatment plans, can be beneficial, excessive reliance on these strategies to manage the disease may lead to increased stress and poorer psychological outcomes.

Emotion-focused coping involves regulating emotions, seeking social support, and reframing thoughts. This approach can provide relief from emotional distress but may not effectively address the underlying issues. Research suggests that using this strategy can improve psychological well-being but may not directly impact disease outcomes. Studies have also indicated that using emotion-focused coping may lead to delays in seeking medical care and non-adherence to treatment.

Smaller studies have found that emotion-focused coping is a more accurate predictor of psychological outcomes than problem-focused coping. For instance, emotion-focused coping is negatively associated with quality of life, perceived health, well-being, and functioning. Additionally, a web-based study of 259 patients with IBD found that self-blame led to more avoidant coping, which was associated with poor adjustment [68].

### 3.3. Interventions in IBD

Berding et al. investigated the favorable consequences of an educational program for individuals with inflammatory bowel disease (IBD). The intervention resulted in a decrease in disease-related anxiety, improved coping with anxiety, heightened satisfaction with disease-related knowledge, and increased use of coping strategies in the intervention group compared to the waitlist group [12]. Moradkhani et al.’s study outcomes further emphasized the notion that individuals with IBD who possess a deeper comprehension of the disease may benefit from it. Those with a greater understanding of IBD were more inclined to adopt adaptive coping strategies. Specifically, participants with higher CCKNOW scores tended to use active coping, planning, instrumental support, and emotional support as coping strategies [25]. These findings are supported by Park et al.’s study, which indicated a negative correlation between higher IBD-KNOW scores and medical acceleration [33]. Additionally, increased disease knowledge leads to better medication adherence, as observed in a cross-sectional survey conducted by Xu et al. Patients with better disease knowledge had higher Morisky scores [34].

Haapamäki et al. investigated the impact of an adaptation course on the health-related quality of life (HRQoL) of patients with IBD. The generic 15-dimensional (15D) tool was used to assess HRQoL, and the Beck Depression Inventory (BDI) was used to evaluate depression. The results demonstrate that scores for 15D, BDI, and psychological well-being showed significant improvements by the end of the course compared to the baseline, and these improvements were maintained during the follow-up period. Notably, marked enhancements in the 15D and BDI scores observed during the course were not related to changes in reported disease activity.

Two studies explored the role of cognitive behavioral therapy (CBT). In the HAPPY-IBD randomized controlled trial led by Stapersma et al., two groups of young individuals were compared as follows: one receiving CBT in addition to standard medical care and the other receiving standard medical care alone. Both groups showed long-lasting improvements in various aspects of their mental and physical well-being, with no significant difference observed between the two groups. Hunt et al. conducted a two-arm randomized controlled trial with a crossover design, in which half of the participants used a CBT-based self-help workbook, while the other half were in an active psychoeducational control group for 6 weeks. Both groups demonstrated improvements in various measures, such as catastrophizing, visceral sensitivity, and HRQL. However, the CBT group generally had larger pre-post effect sizes, and only participants in the CBT group experienced improvements in anxiety and depression.

A randomized controlled trial was conducted to investigate the impact of transcranial direct current stimulation (tDCS) in patients with inflammatory bowel disease (IBD). Volz et al. discovered that tDCS resulted in a significant reduction in global pain, measured using visual analog scale (VAS) scores post-treatment. However, this effect did not persist for one week after treatment. Notably, the active group experienced a significant reduction in pain in the right abdomen one week after tDCS. Additionally, the active group showed a significant decrease in pain catastrophizing compared to the sham group after tDCS, although this effect was not sustained during the one-week follow-up. tDCS also resulted in a short-term reduction in functional symptom severity in IBD patients, with five days of active tDCS treatment. An analysis of disease activity indices (Simple Clinical Colitis Activity Index and Harvey–Bradshaw Index) did not reveal any significant differences at the baseline, post-tDCS, or during one week of follow-up.

Kuo et al. carried out a pilot study at a single center using a single-arm, open-label approach to evaluate the efficacy of RR-MBI (the relaxation response-based mind–body intervention) in treating IBS and IBD. The study reported significant improvements in Pain Catastrophizing Scale scores post-intervention for IBD and during the short-term follow-up for both IBS and IBD. Additionally, Trait Anxiety scores, IBS quality of life, the IBS Symptom Severity Index, and IBD Questionnaire scores demonstrated significant enhancements during the short-term follow-up for both conditions. Notably, RR-MBI resulted in a significant change in the expression of 1059 transcripts in patients with IBD, with approximately 73% of these genes being downregulated. Some genes that experienced reduced expression following RR-MBI are directly linked to inflammatory processes that are crucial in IBD, including several genes involved in interferon regulation and signaling [45].

Numerous studies have explored the advantages of physical activity on the quality of life of individuals with inflammatory bowel disease (IBD), primarily focusing on light-to-moderate exercise. Taylor et al. examined the impact of both walking and moderate-to-vigorous physical activity on physical and mental well-being. Specifically, the study found that moderate-to-vigorous activity had a positive effect on physical health-related quality of life, particularly when performed for more than 150 min per week. Similarly, walking was found to have a greater impact on physical quality of life when practiced for more than 60 min per week. Furthermore, walking for more than 60 min per week, unlike moderate-to-vigorous exercise of any duration, was found to be positively associated with mental health-related quality of life [61].

### 3.4. Relationship between Disease Severity and Coping Strategies

In a cross-sectional study, Jones et al. examined the correlation between the severity of conditions in patients with IBS and IBD and their coping strategies. The study found that, as the severity of the conditions increased, individuals tended to employ a broader range of coping techniques. Specifically, in the case of patients with IBD, seeking social support emerged as a coping strategy with the highest relative score [19].

Additionally, in a study by Kantidakis et al., it was observed that, as the severity of an individual’s IBD symptoms increased, they were more likely to report poorer illness perceptions, reduced self-efficacy, lower quality of life, and heightened psychological distress. The study found that illness perception served as a mediator in the relationship between IBD symptoms, psychological distress, and quality of life [20].

According to Iglesias-Rey et al., individuals without extraintestinal manifestations tend to use emotion-focused coping more frequently, whereas those who have been hospitalized in the past year are more likely to employ the escape–avoidance strategy [18].

In a survey-based study conducted by van Erp et al. on a sample of 245 Dutch patients with IBD (40% male, 73% Crohn’s disease), the direct and indirect effects of disease perception on outcomes and the role of coping strategies as mediating factors were examined. Low illness coherence and high emotional representation had a direct, significant, and unfavorable impact on mental health. On the other hand, high personal control, high consequences (perceived impact on one’s life), and low illness coherence (understanding of the disease) had indirect, significant, and unfavorable effects on the mental health-related quality of life by decreasing activity. In terms of physical quality of life, significant direct effects were found for high consequences and disease activity. However, the indirect effects were similar to those for mental health, with the same factors (high personal control, high consequences, and low coherence) identified, which were again mediated by decreasing physical activity [62].

In their investigation of the impact of resilience on anxiety and depression in a cohort of 228 American patients with IBD (54% female, 89% White, and 58% with Crohn’s disease), Philippou et al. found that high levels of anxiety and depression were associated with the lower odds of having high resilience. However, in the multivariate model, the coefficient for depression was no longer statistically significant. Thus, this study suggests that resilience may have a positive effect on anxiety and depression in patients with IBD.

### 3.5. Stigmatization and Resilience as Modulating Factors for Coping Mechanisms

Patients with IBD frequently encounter social stigmatization, which can lead to discrimination and the exclusion of individuals. The concept of social stigma is multifaceted and comprises the following three domains: enacted stigma (discriminatory experiences), perceived stigma (individuals’ perception of negative attitudes of others), and internalized stigma (conforming to negative social mentality). A study conducted by Taft et al. on 211 patients (156 CD, 55 UC) found that 84% of the participants reported perceived stigma, with no significant differences between UC and CD. The authors did not find any significant differences between patients in remission and those experiencing the exacerbation of symptoms, but individuals with frequent flare-ups reported greater levels of perceived stigma. Greater perceived stigma also correlated with poorer health-related quality of life and was a predictor of reduced compliance with medical treatment [69]. A study published a year earlier by Voth et al. demonstrated that self-blame in patients with IBD is correlated with poorer outcomes and the use of avoidant coping mechanisms [68].

The counterpart of the negative impact of stigma in individuals with IBD is the concept of resilience, which refers to a set of psychological traits that enables adaptive coping. In recent years, research has begun to explore the relationship between resilience and IBD; studies have shown that resilience is positively and significantly associated with health-related quality of life (HRQOL) [61]. It is reasonable to assume that resilience and stigma influence each other, as has been demonstrated for mental health conditions and individuals infected with HIV. A study involving 40 individuals with IBD reported that those with higher resilience scores were more likely to use positive coping mechanisms, such as humor, and were less likely to experience stigma. Stigma was found to be more pronounced in individuals with lower resilience scores, particularly in those with mental health disorders and those lacking support networks [70].

Although the number of studies on resilience in IBD is relatively small compared to other aspects of the condition, there is a growing recognition of the importance of resilience in understanding and managing the psychosocial impact of IBD. The literature review conducted by Lenti et al. reveals that the psychosocial aspects of IBD continue to be given lower priority in comparison to the physical aspects of the disease. Despite the significant impact of stigmatization and resilience on HRQOL and coping mechanisms among these patients, they are insufficiently addressed in clinical practice, which remains an issue [71].

### 3.6. IBD Patients versus General Population

Petrak et al.’s research demonstrated that patients with IBD exhibit a statistically and clinically significant reduction in HRQOL across all subscales of Short-Form 36 (SF-36) compared to the general population. These findings align with those of Graff et al. and Jones et al., who reported that IBD patients had significantly higher scores on measures of psychiatric distress than the control group. Additionally, patients with IBD were found to be less likely to employ coping strategies associated with deliberate problem solving and more likely to use avoidance, confrontative coping, and taking responsibility for their situation compared to the control group. A study conducted by González et al. revealed that individuals with IBD employed distinct coping mechanisms compared with those without the condition. Notably, a significant dissimilarity was observed in the employment of social withdrawal, with IBD patients utilizing this strategy more frequently than healthy subjects. Additionally, significant disparities were observed between emotion-focused engagement coping mechanisms and emotion-focused disengagement. Healthy participants tended to utilize action-oriented coping strategies more frequently than those with IBD, particularly those directed towards adaptive or active solutions. Conversely, healthy subjects engaged less frequently in non-action-oriented coping strategies that focused on emotions [72]. Patients afflicted with IBD utilize a significantly greater number of coping strategies focused on emotional regulation than healthy controls in findings that are consistent with those reported by Iglesias-Rey et al. [18]. Regarding the use of passive or active coping mechanisms, data revealed that individuals in the control group employed passive coping strategies less frequently than patients with IBD. These conclusions are consistent with the previous findings by Grodzinsky et al. [73] and Damopiha et al. [74] in studies on Crohn’s disease patients and healthy controls, respectively.

Coping mechanisms for individuals with IBD may need to adapt to the unpredictable nature of the disease, including periods of remission and flare-ups. This may require flexibility in coping strategies and learning to manage symptoms during periods of increased disease activity. In contrast, coping mechanisms in the general population may become more consistent over time.

Overall, the differences in coping mechanisms between the general population and individuals with IBD reflect the unique challenges and experiences associated with living with chronic illnesses. Understanding these differences can help tailor support and interventions to meet the needs of individuals with IBD.

### 3.7. UC Patients vs. CD Patients

In a multicenter cohort study, Petruo et al. conducted an examination of the psychological distinctions between patients with Crohn’s disease (CD) and those with ulcerative colitis (UC). Their findings indicated that individuals with inflammatory bowel disease (IBD) faced greater psychological challenges when dealing with CD, increased disease activity, and were female. Specifically, CD patients exhibited elevated levels of neuroticism and psychological distress and tended to employ maladaptive coping strategies such as avoidance, along with a reduced need for social support. Conversely, when considering UC patients as a collective group, no discernible differences were observed in the psychological data compared with the control group. However, a more refined analysis revealed psychological impairments in both active CD and active UC patients, as well as in CD patients in remission but not in UC patients in remission [27]. Another distinction observed between the two groups was that patients with CD exhibited a greater inclination towards problem-oriented patterns compared to those with UC [39].

Previous research has shown that patients with IBD do not always seek qualified support immediately after recognizing a flare-up. To investigate the coping strategies used by patients in the context of IBD relapse, Tanaka et al. conducted a survey-based study of 400 Japanese patients, including 260 with UC and 140 with CD. The most common coping methods used by patients with UC were changing the contents of their meals (86%) and getting more sleep (77%) while seeking medical help ranked third (73%). In contrast, patients with CD were more likely to skip meals (81%) and switch to an elemental diet (63%). When asked about the effectiveness of these techniques, patients with Crohn’s disease perceived lifestyle changes (skipping meals, elemental diet) as useful. In contrast, patients with ulcerative colitis tended to use coping methods initially and then switched to increasing the dosage of oral aminosalicylates and topical steroids, which they perceived as more effective. Interestingly, visiting a doctor immediately was not perceived as particularly effective by either group, or the authors cautioned that the wording of the questionnaire may have contributed to this perception.

### 3.8. Active vs. Inactive IBD Patients

According to Gandhi et al., several notable differences were identified between patients with inactive IBD and those with active disease. The former group demonstrated a greater inclination towards participating in their treatment, exhibited a more robust sense of health competence, experienced fewer depressive symptoms, adopted more task-oriented coping strategies, and reported better quality of life in all areas of functioning in relation to IBD. Furthermore, among inactive patients, those who employed more task-oriented coping strategies, including problem-solving and taking action, reported fewer self-reported IBD flare-ups. Moreover, the study revealed that the quality of life among patients with inactive IBD was significantly higher across all four aspects of IBD-related quality of life when compared to those with active disease [15].

### 3.9. IBD and Arthropathies

In a study conducted by van der Have et al. on patients with IBD-associated arthropathy, it was found that back and joint pain had detrimental effects on both quality of life (QOL) and work productivity. Moreover, the study demonstrated a significant connection between emotional representations and the SIBDQ, indicating that lower QOL and greater activity impairments were associated with negative beliefs about how illness impacts emotional well-being [37]. Additionally, a study by van Erp et al. revealed that patients with IBD and arthropathies employed different coping strategies characterized by a greater ability to divert attention and a stronger inclination to contribute to others. They perceived medical interventions as less effective but displayed greater adaptability in managing their daily life activities over time [38].

### 3.10. Youth with IBD

In a study focused on youths with IBD, Reed-Knight et al. found that a higher incidence of family stressors in the preceding 12 months was positively correlated with the expression of pain-related distress. Moreover, greater exposure to family stressors was associated with the increased use of passive coping strategies, which, in turn, was positively linked to elevated levels of depressive symptoms and pain-related distress [28].

Wojtowicz et al. conducted a study that aimed to explore the relationship between pain severity and pain catastrophizing and functional disability in youths with inflammatory bowel disease (IBD). They discovered that a significant proportion of the sample reported experiencing abdominal pain, with 61% of those without clinical disease activity and 75% of those with clinical disease activity reporting some degree of functional disability. A hierarchical multiple regression analysis was conducted to examine the impact of abdominal pain severity and pain catastrophizing on functional disability, controlling for gender. The results show that abdominal pain severity explained 15% of the variation in functional disability scores, while pain catastrophizing, as measured by PCS-C Total Scores, added an additional 7% to the variation in functional disability scores, independent of abdominal pain severity [43].

### 3.11. Children with IBD

Taylor et al. conducted a study examining the differences between IBD and functional abdominal pain in pediatric patients in regard to predictors of depression. In the IBD group, high levels of catastrophizing, disengagement, and withdrawal were associated with increased depression scores, whereas seeking social support displayed an inverse relationship. The most significant predictors of depression in the abdominal pain group were catastrophizing, symptom severity, and seeking social support. Coping strategies exhibited varying effects on disability scores between the two groups. Specifically, symptom severity and catastrophizing were significant factors for IBD patients, while symptom severity, rest, and catastrophizing were significant factors for children with functional abdominal pain [63].

### 3.12. Coping Strategies in CD Patients

McCombie et al. showed that emotion-focused coping was the most prevalent strategy employed by individuals with CD and was associated with worse psychological outcomes [59]. Vigano et al. indicated that denial was more prevalent among patients with CD, who also sought more emotional support [47]. Knowles et al. discovered that disease activity exerted a significant influence on illness perception, which, in turn, had a direct impact on anxiety and depression [53]. Additionally, Knowles et al. highlighted that poor illness perception was observed in CD patients with anxiety, depression, and reduced quality of life [52]. Women demonstrated a greater utilization of emotion-focused, problem-focused, and dysfunctional coping strategies than men [46].

In 2014, Darnopiha explored the relationship between Crohn’s disease activity and perceived stress, coping strategies, and self-efficacy in a sample of 102 CD patients. Statistical analyses demonstrated a significant correlation between the use of maladaptive coping strategies and disease activity [74].

Flett et al. showed that perfectionism is a maladaptive coping factor associated with the amplified impact of IBD [50]. Sense of humor and acceptance were predictors of disease severity [51]. Intrinsic religiousness and spirituality predicted CD remission at a 2-year follow-up [49]. In the case of worsening disease, patients would undergo dietary changes, followed by the addition of an extra drug or seeking help from a doctor [46]. Other authors have suggested that coping has no contributory role when dealing with CD [55,57].

### 3.13. IBD and COVID-19

Lee et al. conducted a study in South Korea during the COVID-19 outbreak to examine the behaviors of IBD patients. It was found that over 80% of patients believed that they should continue their treatment despite nearly half believing that IBD made them more susceptible to infection. The attitudes of the patients towards their treatment differed significantly based on the medication they were taking. Patients taking immunosuppressants and biologics were more likely to be concerned about the increased risk of COVID-19 infection and lung damage than those taking aminosalicylates alone. These patients were also more likely to agree with the statement that IBD patients should avoid hospital visits to reduce their risk of infection [65].

Castellini et al. conducted a survey within the Patient Health Engagement framework of 1014 Italian IBD patients with IBD during the COVID-19 pandemic. The results indicated that patients with higher engagement reported higher coping self-efficacy, lower stress perception, a reduced perception of infection risk, and a higher sense of responsibility in preventing infection. This suggests a more adaptive coping approach for patients with higher engagement [67].

Prioritizing mental health through stress-management techniques, open communication with healthcare providers, and finding virtual support groups were crucial elements of coping during this challenging time [75].

## 4. Discussions

IBD poses a significant challenge to affected individuals and has a profound impact on their quality of life and overall well-being. As the prevalence of IBD continues to increase globally, it is crucial to understand effective coping strategies to optimize patient outcomes. This narrative review aimed to comprehensively assess and synthesize the existing literature on the coping strategies employed by individuals with IBD. The synthesis of these findings not only highlights the diverse coping mechanisms used by individuals with IBD but also emphasizes the importance of tailored interventions in the management of the disease.

The variety of coping strategies observed across different studies underscores the need for a nuanced understanding of individual differences and inherent heterogeneity within IBD populations. Factors such as disease severity, disease duration, and individual psychosocial characteristics may influence the selection and effectiveness of coping mechanisms [76]. Therefore, tailoring interventions to the specific needs of patients is crucial for enhancing coping effectiveness and overall well-being [77].

Patients with IBD frequently withdraw from social situations to avoid discomfort. Nevertheless, social support is vital for individuals with chronic conditions as it improves treatment adherence and adaptation to illness. These patients tend to employ passive coping strategies that focus on emotions, which require less psychological effort but result in greater psychological discomfort and poor adjustment to disease. Therefore, it is imperative to encourage patients to expand their social networks and interact with others, including those with IBD. Additionally, those who rely on passive and emotion-focused strategies should be taught and encouraged to adopt active and problem-focused approaches to facilitate better adaptation to the disease. There is a need to investigate additional psychological factors that may influence the coping mechanisms employed by these patients, such as stigmatization and resilience. Resilience can be developed through targeted interventions, which, in turn, can enhance an individual’s ability to cope with their illness [78].

Incorporating interventions that enhance self-esteem and provide patients with the necessary skills to effectively manage stressful situations is crucial. In doing so, patients may no longer perceive their circumstances as threatening, which may lead to a reduction in the frequency of exacerbations and fewer hospitalizations. This, in turn, can contribute to the more effective management of illnesses [79].

The potential benefits of incorporating technology and digital health interventions into coping strategies for IBD warrant further exploration in both research and clinical practice. Mobile applications, virtual support groups, and telemedicine platforms have the potential to enhance access to coping resources, facilitate remote monitoring, and foster a sense of community among individuals with IBD. However, it is essential to conduct rigorous evaluations and the validation of these interventions to ensure their efficacy and safety in the specific context of IBD management [77].

The literature on coping with IBD is characterized by inconsistency, which can be attributed to differences in the research design and coping instruments used. Numerous studies in this area are cross-sectional and involve mixed IBD groups at different stages of their illness. Furthermore, the observational design of many studies included in our review precludes us from confirming or denying a causal relationship between the assessed parameters. Additionally, the cross-sectional nature of these studies prevents the establishment of a temporal relationship, while case–control studies may be susceptible to selection bias.

Selection bias presents a significant challenge in studies examining coping mechanisms within the context of IBD. Individuals who volunteer for studies on coping may already have a greater awareness of their own coping styles and might be more motivated to manage their condition compared to the general IBD population. This could lead to an overrepresentation of those with healthier coping mechanisms, skewing results and misrepresenting the full range of coping styles used by IBD patients. Furthermore, participants with particularly severe symptoms or those currently experiencing flare-ups might be less likely to enroll in such studies, potentially underestimating the negative impact of maladaptive coping on disease outcomes.

Our review emphasizes the scarcity of longitudinal studies exploring the dynamic nature of coping strategies for IBD over time. Such investigations are crucial for understanding how coping mechanisms evolve with disease progression and in response to life events, thereby enabling the development of adaptive and timely interventions. We recommend that future research prioritize longitudinal studies to elucidate the temporal dynamics of coping in IBD populations.

It is important to recognize certain limitations of our review, which provides a comprehensive overview of the coping strategies for IBD. The variability in measurement tools and outcome assessments across studies poses challenges for conducting direct comparisons and meta-analyses. To facilitate more robust comparisons and enhance the generalizability of the findings, it is essential to standardize measurement instruments and outcome measures in future research endeavors.

Effective communication between healthcare providers and patients is crucial for building a strong doctor–patient relationship. To achieve this, it is essential to recognize the coping mechanisms used by patients. Research has shown that patients who rely on maladaptive coping mechanisms tend to perceive their physicians as being disengaged and unsupportive. The relationship between maladaptive coping mechanisms and negative mental health outcomes is well established. Numerous studies have demonstrated that avoidance-based coping styles are correlated with psychiatric disorders, such as post-traumatic stress disorder (PTSD), anxiety, and major depression [80].

Cross-sectional studies are inadequate to evaluate the predictive capabilities of different coping strategies. Longitudinal and interventional studies are necessary to provide a more comprehensive understanding of the relationship between coping strategies and psychological outcomes. To assess the effectiveness of various coping strategies, it is necessary to develop a specific coping instrument tailored to patients with inflammatory bowel disease. If a clinically significant association exists between coping and psychological outcomes, enhancing adaptive coping behaviors and reducing maladaptive coping behaviors may lead to improved outcomes in IBD patients. As a holistic approach to managing IBD patients continues to progress, understanding the role of coping strategies becomes increasingly critical for achieving optimal patient care.

Most studies included in the review were cross-sectional in nature, which limited our ability to make inferences about causation or assess the long-term effects of coping strategies on disease outcomes. Future research should prioritize well-designed longitudinal studies with larger sample sizes to elucidate the causal relationships between coping strategies and clinical outcomes in IBD.

## 5. Conclusions

People who have been diagnosed with IBD often experience a chronic and unpredictable condition that can significantly impact their physical, psychological, and social well-being due to ongoing changes. Based on the outcomes and constraints of the studies included in this review, several conclusions can be drawn. The results indicate that passive strategies are the predominant coping response among individuals with depressive symptoms, while active strategies are the most effective at enhancing physical functioning. Thus, it is crucial to delve deeper into how coping mechanisms may affect the perception of symptoms over time. Incorporating an assessment of patients’ coping strategies as part of a comprehensive multidisciplinary treatment plan may also help identify those at the highest risk of poor adjustment to the disease. The influence of coping responses on general quality of life appears to be important. Given the diversity of IBD, future studies ought to explore the intricate relationship between age, disease progression, and coping with symptoms in greater detail.

## Figures and Tables

**Table 1:** Summaries of studies examining coping techniques in IBS.

Author (Year)	Study Design and Participant Characteristics	Coping Measures/Other Outcome Measures	Impact on HRQoL	Findings
Luo et al. [11]; (2018)	Prospective cohort study; 229 UC pts, male/female: 116/113, mean age 40.4 years.	PSS, MCMQ, IBDQ	A higher PSS score and acceptance–resignation behavior were associated with poorer HRQol	The presence of acceptance–resignation behavior was linked to diminished quality of life and unfavorable prognosis among UC patients.
Berding et al. [12]; (2017)	Prospective, randomized, waitlist-controlled trial; 181 IBD pts, male/female: 56/125, mean age 39.9 years.	IBDPC, FoP-Q-SF, heiQ, GIBDI, SF-12, PHQ-4	The effects of education on HRQoL could not be shown.	The patient education seminar had a positive impact on IBD patients, improving their knowledge and ability to cope with anxiety. At the three-month mark, patients used significantly more coping strategies.
Chao et al. [13]; (2019)	Cross-sectional study; 207 IBD pts, male/female: 88/119, mean age 39 years.	SIBDQ, IBDDI, WPAI, HADS, Brief COPE, GSESP, PMS, HBI	Disease activity and maladaptive coping were linked to diminished quality of life.	Maladaptive coping and disease activity were individually associated with unfavorable PRO (patient-reported outcomes). Higher self-efficacy in patients was positively associated with adaptive coping mechanisms.
Freitas et al. [14]; (2015)	Cross-sectional study; 147 IBD pts, male/female: 63/84, mean age 45.1 years.	Brief COPE Scale, HADS, WHOQOL-Bref, MMAS-8	Depression and anxiety were found to be independent predictors of HRQoL.	Religious struggle was linked to psychological distress symptoms and lower treatment adherence, while positive religious coping had a favorable impact on satisfaction with health.
Gandhi et al. [15]; (2016)	Cross-sectional study; 70 IBD pts, male/female: 29/41, mean age 43.26 years.	IBDQ, BDI, PHCS, CISS	Patients with active disease had significantly worse quality of life compared to inactive IBD patients.	Inactive patients were more likely to practice task-oriented coping, compared with active IBD patients. Active coping styles were also associated with a lower risk of relapse.
Graff et al. [16]; (2009)	Cross-sectional study; 356 IBD pts, male/female: 150/206, mean age 38.5 years for CD pts and 43.0 years for UC pts.	The Mastery Scale and The Psychological Well-being Manifestations Scale, K-10	Not evaluated.	A lower mastery and psychological well-being and a higher distress level were observed in the IBD sample in comparison with the non-IBD controls. Maladaptive coping approaches were more likely to be used by patients with active IBD.
Haapamäki et al. [17]; 2018	Cohort study; 142 IBD pts, male/female: 40/102, mean age 43.4 years.	15D, BDI, Ojanen’s scales	Patients who benefited from peer support experienced better quality of life at the 12-month follow-up.	The adaptation courses had a positive impact on the 15D and BDI scores, which reflect the improved well-being of the patients. Many of the respondents considered the gain of peer support as the most beneficial aspect of the course.
Iglesias-Rey et al. [18]; (2012)	Prospective transversal study; 799 IBD pts, male/female: 377/422, mean age 44.63 years.	HADS, PSS, COPE Inventory	Not evaluated.	The most frequent type of coping among IBD patients was emotion-focused coping. This coping strategy was associated with higher scores for anxiety.
Jones et al. [19]; (2006)	Cross-sectional study; 48 IBD pts, male/female: 20/28, mean age 39 years.	IBS-QOL, IBDQ, SCL-90-R, WOC, ISEL, TAS-20, SAS	In terms of quality of life, there was no significant difference between IBD and IBS patients. In comparison with controls, IBD and IBS patients had a poorer HRQOL.	Patients suffering from IBD were less inclined to embrace coping techniques centered around strategic problem-solving and optimistic reassessment compared to their healthy counterparts.
Kantidakis et al. [20]; (2021)	Cross-sectional study; 261 IBD pts, male/female: 63/198, mean age: 37 years.	Brief-COPE, BIPQ, MAAS, NGSE, DASS-21, IBDQ	When an individual’s IBD activity intensifies, they tend to report a decline in their quality of life.	The connections between IBD symptoms and both depression and anxiety, as well as between IBD symptoms and quality of life, are statistically mediated through psychological factors, such as maladaptive coping and illness perceptions.
Knowles et al. [21]; (2013)	Preliminary study; 83 IBD pts, male/female: 23/60, mean age 38.48 years.	HSS, BIPQ, Brief-COPE, HADS	Not evaluated.	A less favorable perception of illness was associated with a greater tendency to employ maladaptive coping strategies, which, in turn, had a negative effect on anxiety and depressive symptoms.
McCombie et al. [22]; (2016)	Methodological study; Sample A: 199 IBD pts, male/female: 44/155, mean age 38.8 years. Sample B: 58 IBD pts, male/female: 15/43, mean age 44.3 years. Sample C: 179 IBD pts, male/female: 82/97, mean age 38.3 years.	IBD-Cope, Brief COPE, IBDQ, HADS, SFQ	Elevated scores in maladaptive coping were linked to reduced HRQol health.	The IBD-Cope is a compact questionnaire tailored to inflammatory bowel disease (IBD) patients, and it has been proven to be both reliable and valid.
McCombie et al. [23]; (2015)	Prospective observational study; 54 IBD pts, male/female: 27/27, mean age 33.5 years.	SIBDQ, SF-12, Brief COPE, EPQ-BV, HADS	Significant improvement in HRQOL was observed at the 6-month follow-up after the diagnosis.	The use of maladaptive coping strategies is linked to more pronounced psychological anxiety, depression, and poorer physical outcomes in terms of health-related quality of life (HRQOL).
Minderhoud et al. [24]; (2004)	107 IBD pts, male/female: 51/56, mean age 42.8; 66 controls, male/female: 34/32, mean age 45.5 years.	IBDQ, CISS, The Manning Criteria, The Rome II Criteria	The presence of IBS-like symptoms had a detrimental impact on the quality of life of patients.	No correlation was detected between the presence of IBS-like symptoms and coping strategies.
Moradkhani et al. [25]; (2011)	Cross-sectional study; 111 IBD pts, male/female: 25/86, mean age 31 years.	CCKNOW, Brief COPE scale, MMAS-4	Not evaluated.	A positive correlation was identified between a higher level of IBD knowledge and increased scores in active coping.
Petrak et al. [26]; (2001)	Cross-sectional study; 1319 IBD pts, male/female: 631/688, mean age 39.6 years.	SF-36, FKV-LIS, H-RB-Skala	In comparison to the general population, there is a statistically significant reduction in HRQoL.	Active coping negatively impacts patients’ overall physical HRQOL during an active phase, whereas this relationship is absent in the case of patients in remission.
Petruo et al. [27]; (2019)	Multicenter cohort study; 110 CD pts, male/female: 38/72, mean age 35.07 years; 67 UC pts, male/female: 29/38, mean age 35.76 years; 120 controls, male/female: 41/79, mean age 36.92 years.	SCL-90-R, SVF 78, HPI	Not evaluated.	Patients with Crohn’s disease (CD) experienced a greater degree of psychological impairments in comparison to individuals with ulcerative colitis (UC) or the control group.
Reed-Knight et al. [28]; (2018)	Cross-sectional study, 183 IBD pts, male/female: 96/87, mean age 13.75 years; 183 parents, male/female: 18/165, mean age 44.38 years.	The Affective Distress subscale from the PBCL, FILE, PRI, CDI	Not evaluated.	Increased family stress correlated with heightened utilization of passive coping strategies and higher levels of depressive symptoms.
Reed et al. [29]; (2021)	Cross-sectional study; 147 IBD pts, male/female: 76/71, mean age 13.88 years.	Global Self-Worth subscale from the SPPC, PRI, The Passive Coping Scale, IMPACT-III	Youths who indicated a higher reliance on passive coping techniques reported a lower HRQoL.	The overall self-esteem of pediatric patients can influence their HRQOL by way of passive coping strategies, particularly through maladaptive cognitions like catastrophizing.
Rudnik et al. [30]; (2019)	Preliminary report; 33 IBD pts, male/female: 19/14, mean age 35.3 years.	FCSQ-14, CFI, SWLS	Stress coping and cognitive flexibility lead to higher HRQoL.	Cognitive flexibility and adaptability in handling stress are related to health-related quality of life.
Scott et al. [31]; (2020)	Qualitative study; 12 IBD pts, male/female: 5/7.	SIBDQ	Not evaluated	Coping strategies in African American IBD patients were similar to those in other IBD populations.
Stapersma et al. [32]; (2020)	Randomized controlled trial; PASCET-PI group: 37 IBD pts, male/female: 10/27, mean age 18.62 years; CAU group: 33 IBD pts, male/female: 12/21, mean age 17.69 years.	SCARED, HADS-A, CDI, BDI-II, IMPACT-III, IBDQ, CERQ, B-IPQ, sleep problem items of the YSR and ASR, sPC-DAI, PUCAI	Both groups of young individuals showed enhanced HRQoL up to the 12-month final follow-up assessment.	Across a span of 12 months, young individuals in both groups experienced enhancements in their psychological outcomes, including anxiety, depression, coping, and illness perceptions.
Park et al. [33]; (2020)	Cohort study; 298 IBD pts, male/female: 207/92, mean age 39.8 years.	IBD-KNOW questionnaire	Not evaluated.	Greater IBD-KNOW scores are inversely related to medical acceleration.
Xu et al. [34]; (2022)	Cross-sectional survey; 105 IBD pts, male/female: 67/38, mean age 32.79 years.	MMAS	Not evaluated	The Morisky score was significantly influenced by factors such as medication frequency, the method of medication administration, and the level of disease understanding.
Sirois et al. [35]; (2017)	Prospective cohort; T1: 420 IBD pts, male/female: 100/320, mean age 35.4 years; T2: 152 IBD pts, male/female: 34/118, mean age 37.9 years.	The Psychological Thriving Scale Acceptance subscale of the ICQ, coping efficacy scale, CES-D, the Duke–UNC Functional Social Support Questionnaire	Not evaluated.	Approaches focusing on coping enhancement and the management of depressive symptoms could enhance thriving in the context of IBD.
Sheehan et al. [36]; (2023)	Cross-sectional study, 160 IBD pts, male/female: 72/86.	IBD-SES, CD-PRO, UC-PRO	Concentrating on improving self-efficacy domains could potentially enhance overall HRQol.	Increased confidence in handling stress, and emotions, as well as managing symptoms and the disease, was linked to a reduction in the impact of IBD on daily life.
van der Have et al. [37]; (2015)	Prospective cohort study; 204 IBD pts, male/female: 82/122, mean age 44.3 years.	IPQ-R, CORS, SIBDQ, SF-36, WPAI	Negative beliefs regarding the impact of the illness on emotional well-being were linked to diminished HRQoL.	After accounting for disease activity, and back and joint pain, both illness perceptions and coping techniques emerged as notable predictors of both QOL and work productivity.
van Erp et al. [38]; (2018)	Prospective cohort study; 123 IBD pts with arthropaties, male/female: 40/83, mean age 44.1 years; 81 IBD pts without arthropaties, male/female: 43/38, mean age 44.5 years.	IPQ-R, CORS, SF-36, WPAI	Patients with arthropathies exhibited a lower HR QoL when compared to patients without arthropathies.	Patients with IBD and arthropathies showed a higher level of symptom-related concerns and decreased understanding of IBD compared to patients without arthropathies.
Kinash et al. [39]; (1993)	Cross-sectional study; 150 IBD pts, male/female: 78/72.	JCS, EPQ, BDI	Not evaluated	IBD patients exhibited notably higher scores in employing problem-oriented coping strategies compared to affective-oriented methods.
Hunt et al. [40]; (2019)	Randomized controlled trial; 140 IBD pts, male/female: 48/92, mean age 35.64 years.	HBI, GSRS, VSI, GI-COG, SIBDQ, STAI, BDI	The CBT group significantly outperformed the PE group in improving HRQL.	Scores on catastrophizing, visceral sensitivity, and HRQL improved in both the CBT and PE groups. Anxiety and depression saw significant improvements solely among CBT group participants.
van Erp et al. [41]; (2021)	Cross-sectional study; 686 IBD pts, male/female: 280/406, mean age: 50 years.	IBDQ	IBD patients who demonstrated elevated levels of acceptance and/or perceived control experienced superior HRQoL.	The validity of the segmentation model, which relies on disease acceptance and perceived control, was confirmed in IBD patients.
Wang et al. [42]; (2021)	Cross-sectional study; 135 IBD pts, male/female: 93/42, mean age 32.62 years.	CD-RICS, FFMQ, GWB	The general well-being of IBD patients was observed to be lower compared to the ordinary model.	Psychological resilience was found to act as a mediator between mindfulness levels and overall well-being.
Wojtowicz et al. [43]; (2014)	Cross-sectional study; 75 IBD pts, male/female: 41/34, mean age 14.5 years.	PCS-C, FDI, API	Not evaluated.	Pain catastrophizing added a significant 7% to the prediction of functional disability, independently of pain severity.
Volz et al. [44]; (2016)	Randomized controlled trial; 20 IBD pts, male/female: 7/13, mean age: 37.5 years	Edinburgh Handedness Inventory, BDI, the German version of the Pain Catastrophizing Scale, IBDQ, IBS-SSS	Examinations of the IBDQ did not show any notable distinctions at the baseline, post-stimulation, or during the subsequent analysis one week after the stimulation.	Pressure pain threshold measurements did not exhibit any statistically significant differences between pre- and post-intervention values. One week after concluding the stimulation, the active group continued to show reduced pain on the right side of the abdomen.
Kuo et al. [45]; (2015)	Uncontrolled pilot study; 29 IBD pts, male/female: 12/17, mean age 40.5	STAI-Y, PCS, BPI, IBS-QOL, IBS-SSI, IBD-Q	RR-MBI has demonstrated effectiveness in enhancing the HRQoL for individuals with both IBS and IBD.	RR-MBI led to changes in the expression of a greater number of genes in IBD (1059 genes) compared to IBS (119 genes). In IBD, the reduced expression of genes influenced by RR-MBI was associated with pathways related to inflammatory response.
Tanaka et al. [46]; (2009)	76 CD pts, male/female: 61/15, mean age 35.3 years.	Patients’ coping measures in worsening disease conditions	Not evaluated.	Dietary changes were most consistent among patients (70%), followed by taking extra medicine (42%) or seeing a doctor immediately (20%).
Vigano et al. [47]; (2016)	Prospective study, 95 CD pts, male/female: 57/38, mean age: 45 years.	HADS, CBA, BDI, Brief-COPE	Not evaluated.	Positive reframing and planning were lower in CD patients with depression. Denial and seeking emotional support were higher in these patients.
Sarid et al. [48]; (2017)	Cohort study; 402 CD pts, male/female: 158/244, mean age: 36.5/40.1 years.	HBI, Brief COPE Inventory, SWLS, SIBDQ	QoS scores were higher in men than women with active disease. Economic status, dysfunctional coping, and the number of children impacted HRQoL.	Women showed more use of emotion-focused, problem-focused, and dysfunctional coping strategies than men. In patients with active disease, SWLS and SIBDQ scores were reduced.
De Campos et al. [49]; (2021)	90 CD pts, male/female: 39/51, mean age 39.3 years.	HBI, IBDQ, HADS, DUREL, SSRS	There was no significant association of religiousness or spirituality with HRQoL in this sample	Intrinsic religiousness and spirituality predicted the remission of CD at a 2-year follow-up.
Flett et al. [50]; (2011)	Cross-sectional study, 51 IBD pts, male/female: 20/31, mean age 37.7 years.	PSPS, SIP136, CHIP, LOT	Not evaluated.	Perfectionism is a maladaptive coping factor associated with an amplified impact on IBD.
Friger et al. [51]; (2014)	192 CD pts, male/female: 81/111, mean age 36.8 years.	HBI, SF-36 QoL, Ways of Coping	Low SF-36 score as a predictor of disease severity	Sense of humor and acceptance were predictors of disease severity.
Knowles et al. [52]; (2013)	31 CD pts, male/female: 17/14, mean age 45 years.	BIPQ; HADS, SQOL	Insufficient evidence	Poor illness perception was shown in patients with anxiety, depression, and reduced quality of life.
Knowles et al. [53]; (2011)	Cross-sectional questionnaire-based study, 96 CD pts, male/female: 34/62, mean age: 38 years.	CDAI, Carver Brief coping scale, BIPQ, HADS.	Not evaluated.	Disease activity has an increased influence on illness perception. Illness perception has a direct influence on anxiety and depression.
Larsson et al. [54]; (2016)	Qualitative study, 15 IBD pts.	Interviews to assess disease-related stress, coping strategies, and the need for information	Not evaluated.	The patients adopted behavioral, social, and emotional strategies to cope with stress.
Van der Have et al. [55]; (2013)	Cross-sectional study, 82 CD pts, male/female: 30/52, mean age 42 years.	IPQ-R, Utrecht Coping List, HRQOL	Impacted by disease activity, self-perceived health, and perceived consequences.	Coping has no contributory role.
Zhang et al. [56]; (2018)	Prospective study, 82 CD pts, male/female: 45/37.	HBI, BIPQ, BCOPR, HADS, QoL	Significant improvement in HRQoL after treatment.	Maladaptive coping is correlated with patients’ physical and psychological status and decreases significantly after effective treatment.
Dorrian et al. [57]; (2009)	80 CD pts, male/female: 37/43, mean age 40 years.	CDAI, SF-MPQ, IPQ-R, COPE	Not evaluated.	Coping strategies do not affect quality of life once illness perception is controlled.
Parekh et al. [58]; (2015)	Cross-sectional study, 150 CD pts, male/female: 77/73, mean age 39.3 years.	QOL, SIBDQ, JCS	Patients with an increased number of flares have lower HRQoL.	The primary mechanisms identified were confronting, evasive, optimistic, and fatalistic.
McCombie et al. [59]; (2013)	Systematic review of 39 articles.	Coping mechanisms	Not evaluated.	The most common coping mechanism was emotion-focused and was associated with worse psychological outcomes. Problem-focused coping was consistently associated with better psychological outcomes.
Tanaka et al. [60]; (2016)	Cross-sectional study, 260 UC pts, male/female: 130/130, mean age 47.2 years; 140 CD pts, male/female: 95/45, mean age 41.3 years.	Questionnaire	Not evaluated.	UC: the changing content of meals is most popular, and increasing aminosalicylates or steroids is usually required. CD: skipping meals and an elemental diet are the most popular. Dietary changes are more effective for CD
Taylor et al. [61]; (2018)	Cross-sectional study, 242 IBD pts, male/female: 48/194, mean age 39.6 years.	International Physical Activity Questionnaire short version, SF-36, CD-RISC	Physical HRQoL exhibited a positive correlation with moderate physical activity, resilience, and walking.	Walking (>60 min/week) increases mental QoL; Moderate–vigorous physical activity increases physical health-related QoL
van Erp et al. [62]; (2017)	Cross-sectional study, 211 IBD pts, male/female: 84/127, mean age: 42.9 years.	IPQ-R, CORS, SF-36, WPAI	Illness perceptions and coping exerted an influence on HRQoL.	Psychological and behavioral factors perceived as causes of IBD; mental QoL is negatively and directly affected by low illness coherence and high emotional representation. Physical QoL is negatively and directly affected by high consequences (i.e., perceived impact) and disease activity
van Tilburg et al. [63]; (2015)	Cross-sectional study, 189 IBD pts, male/female: 97/92, mean age 13.7 years.	PRI, IBDS, FDI, CDI	Not evaluated	Depression scores lower in IBD; catastrophizing, disengagement, and withdrawal increase depression scores, seeking social support, and decreasing depression in IBD. Symptom severity and catastrophizing are associated with higher disability in IBD
Philippou et al. [64]; (2022)	Cross-sectional study, 288 IBD pts, male/female: 104/124, mean age 35.0 years.	CD-RISC, PHQ-9, GAD-7	Not evaluated	Resilience is negatively associated with either anxiety or depression (bivariate model), but only anxiety was significantly associated in the multivariate model
Lee et al. [65]; (2022)	Cross-sectional study, 544 IBD pts, male/female: 206/338, mean age 40.34 years.	Telephone surveys	Not evaluated	Over 50% worried about higher infection risk due to treatment, but over 80% considered that they should continue. Patients on immunosuppressants and biologics were more worried than patients on aminosalicylates; The majority did not discuss their symptoms with a specialist, and 7% discussed interrupting medication
Popa et al. [66]; (2022)	Case-control study, 70 IBD pts, male/female: 26/44, mean age 38.03 years.	PSS, COPE questionnaire, SF-36 scale	There was no correlation observed between potential risk factors and HRQoL. Partea superioară a formularului	Higher stress and lower QoL (excepting emotional role functioning) in the IBD group; Problem-focused and emotion-focused coping is preferred over maladaptive coping and alcohol/substance abuse
Castellini et al. [67]; (2022)	Cross-sectional study, 1014 IBD pts.	PHE scale	Not evaluated	Higher engagement is associated with higher coping self-efficacy, lower stress perception, lower perceived infection risk, and higher responsibility in preventing infection

15D: the generic 15-dimensional tool; API: the abdominal pain index; ASR: Adults Self-Report; B-IPQ: Brief Illness Perceptions Questionnaire; BCOPR: Brief Coping Operations Preference Enquiry; BDI: Beck’s Depression Inventory; Brief RCOPE: Brief Religious Coping inventory; CBA: Cognitive Behavioral Assessment; CDI: Children’s Depression Inventory; CD-PRO: Crohn’s Disease Patient-Reported Outcome; CD-RICS: Connor–Davidson Resilience Scale; CCKNOW: Crohn’s and Colitis knowledge score; CES-D: Center for Epidemiological Studies Depression; CERQ: Cognitive Emotion Regulation Questionnaire; CHIP: Coping with Health Injuries and Problems Scale; CISS: Coping Inventory for Stressful Situations; CORS: Coping with Rheumatic Stressors Questionnaire; DASS-21: Depression, Anxiety and Stress Scales; DUREL: Duke University Religion Index; EPQ: Eysenck Personality Questionnaire; EPQ-BV: The Eysenck Personality Questionnaire—Brief Version; FCSQ-14: Flexibility in Coping with Stress Questionnaire; FILE: The Family Inventory of Life Events and Changes; FDI: The Functional Disability Inventory; FKV-LIS: Freiburger Fragebogen zur Krankheitsverarbeitung; FoP-Q-SF: Fear of Progression Questionnaire—Short Form; GAD-7: Generalized Anxiety Disorder 7; GIBDI: German Inflammatory Bowel Disease Activity Index; GI-COG: Gastrointestinal Cognitions Questionnaire; GISS: Gastrointestinal Symptom Scale; GSRS: Gastrointestinal Symptom Rating Scale; GWB: General Well-Being Schedule; HADS: Hospital Anxiety and Depression Scale; HBI: Harvey Bradshaw index; H-RB-Skala: Scale for the Assessment of Hopelessness; HSS: Health Status Subscale; IBD-SES: IBD Self-Efficacy Scale; IBDDI: IBD disability index; IBDS: Inflammatory Bowel Disease Symptom Questionnaire; IBDQ: Inflammatory Bowel Disease Questionnaire; IBDPC: IBD Patient Concerns questionnaire; ICQ: Illness Cognition Questionnaire; ISEL: The Interpersonal Support Inventory List; JCS: Jalowiec Coping Scale; K-10: Kessler Psychological Distress Scale; LOT: Life Orientation Test; MAAS: Mindful Attention Awareness Scale; MCMQ: Medical coping modes questionnaire; MMAS-8: Morisky Medication Adherence Scale; MPQ: McGill Pain Questionnaire, Short Form; NGSE: New General Self-Efficacy Scale; PHE scale: Patient Health Engagement; PHCS: Perceived Health Competence Scale; PHQ-4: The Patient Health Questionnaire-4; PHQ-9: The Patient Health Questionnaire-9; PRI: Pain Response Inventory; PSPS: Perfectionistic Self-Presentation Scale; PUCAI: the Pediatric Ulcerative Colitis Activity Index; PSS: Perceived Stress Scale; QSOL: Stoma Quality of Life Scale; SCL-90-R: Symptom Checklist-90-revised; SCARED: Screen for Child Anxiety-Related Emotional Disorders; SF-12: Short Form Health Survey; SFQ: Social Functioning Questionnaire; SIBDQ: Short Inflammatory Bowel Disease Questionnaire; SIP136: The Psychosocial Impact Subscale of the Sickness Impact Profile; SPPC: The Self-Perception Profile for Children; SQOL: Stoma Quality of Life Scale; SSRS: Social Skills Rating System; STAI: Spielberger State Trait Anxiety Inventory; STAI-Y: State-Trait Anxiety Inventory; TAS-20: Toronto Alexithymia Scale; UC-PRO: Ulcerative Colitis Patient Reported Outcome; VSI: Visceral Sensitivity Index; WHOQOL-Bref: World Health Organization Quality-of-Life Scale; WOC: Ways of Coping Questionnaire; WPAI: The Work Productivity and Activity Impairment questionnaire; YSR: Youth Self-Report.
